# Nomogram to predict overall survival of patients receiving radical gastrectomy and incomplete peri-operative adjuvant chemotherapy for stage II/III gastric cancer: a retrospective bi-center cohort study

**DOI:** 10.1186/s12885-024-12103-1

**Published:** 2024-03-18

**Authors:** Dian Liu, Hu Quan, Min Ma, Huijun Zhou, Xiaolin Yang, Zhengchun Wu, Jia Luo, Hua Xiao, Yanping Xiao

**Affiliations:** 1 Department of Lamphoma and Abdominal Radiotherapy, Hunan Cancer Hospital and the Affiliated Cancer Hospital of Xiangya School of Medicine, Central South University, 410013 Changsha, China; 2 Department of Hepatobiliary and Intestinal Surgery, Hunan Cancer Hospital and the Affiliated Cancer Hospital of Xiangya School of Medicine, Central South University, 410013 Changsha, China; 3 Department of Gastrointestinal Surgery, The Third Xiangya Hospital of Central South University, 410013 Changsha, China; 4 Department of Gastroenterology and Urology, Hunan Cancer Hospital and the Affiliated Cancer Hospital of Xiangya School of Medicine, Central South University, 410013 Changsha, China; 5 Department of Gastroduodenal and Pancreatic Surgery, Affiliated Cancer Hospital of Xiangya School of Medicine, Hunan Cancer Hospital, Central South University, 410013 Changsha, China; 6 Department of Scientific Research, Changsha Health Vocational College, 410605 Changsha, China

**Keywords:** Gastric cancer, Adjuvant chemotherapy, Survival, Nomogram, Validation

## Abstract

**Background:**

To establish a nomogram to predict the probability of survival of patients with stage II/III gastric cancer (GC) who received incomplete peri-operative adjuvant chemotherapy (PAC).

**Methods:**

The medical records of stage II/III GC patients who received curative resection and 1 to 5 cycles of PAC from two tertiary hospitals were retrospectively reviewed. Patients were randomly classified into either a training group or validation group at a ratio of 7:3. The nomogram was constructed based on various prognostic factors using Cox regression analysis in the training cohort, and was validated by the validation group. Concordance index and calibration curves were used to evaluate the discrimination and calibration of the nomogram. Additionally, decision curve analysis (DCA) was used to compare the net clinical benefits of the nomogram and eighth version of TNM staging system.

**Results:**

A total of 1,070 consecutive patients were included and 749 patients were enrolled into the training group. Lower body mass index (< 18.5 kg/m^2^), total gastrectomy, stage III disease and fewer cycles of PAC were identified to be independent predictors for poorer survival. The area under the curve (AUC) values of receiver operating characteristics (ROC) curve predicting 5-year survival probabilities and C-index were 0.768 and 0.742, 0.700 (95%CI: 0.674–0.726) and 0.689 (95%CI: 0.646–0.732) in the training and validation groups, respectively. The calibration curves in the validation cohort showed good agreement between the prediction and observation of 1-, 3- and 5-year survival probabilities. Furthermore, DCA showed that our model has a better net benefit than that of TNM staging system.

**Conclusions:**

The findings emphasize the value of completing PAC. The nomogram which was established to predict survival probability in patients with stage II/III GC receiving radical gastrectomy and incomplete PAC had good accuracy and was verified through both internal and external validation.

**Supplementary Information:**

The online version contains supplementary material available at 10.1186/s12885-024-12103-1.

## Introduction

Gastric cancer (GC), with an estimated incidence of over one million new cases in 2020, is one of the most common malignancies and gastrectomy offers the only possible curative treatment to date [1]. Unfortunately, only about a quarter of GC patients were diagnosed at an early stage in the West and China. For those patients with locally advanced gastric cancer (LAGC), long term outcomes remain unsatisfactory even after curative resection, with nearly 40% of patients experiencing relapse within 2 years after their operations [2]. To reduce the recurrence rate, peri-operative adjuvant chemotherapy (PAC), including neo-adjuvant chemotherapy (NAC) and/or adjuvant chemotherapy (AC), is a well-established management regimen, in addition to curative resection for stage II/III GC [3–4]. Unfortunately, few patients received sufficient cycles of PAC in clinical practice, even during recent prospective large scale phase 3 research trials [5–7]. The possible explanations include the poor general conditions of patients, those suffering from complications after their operation, or severe toxicity induced by chemotherapy.

Although several predictive models have been established to assess the survival probability of GC patients, in most of these patients were usually roughly divided into those who received or did not receive AC [8–11]. Unfortunately, the exact cycles of PAC have rarely been clearly reported in the literature. Given that the relative dose intensity has been shown to be significantly associated with prognosis in several types of malignancies [12–14], it is reasonable to hypothesize that the potential predictors may be different in stage II/III GC patients who had received inadequate or adequate PAC. Moreover, the exact cycles of PAC may also be related to the long term outcomes for these patients. Therefore, in this retrospective study conducted in two tertiary hospitals in China, for the first time, we investigated whether the exact cycles of PAC significantly impacted overall survival (OS) of these patients. In addition, the aim was to develop a novel nomogram to predict survival probability of patients with stage II or III GC who had received curative resection but inadequate PAC.

## Methods

### Patients

The medical records of 2,805 consecutive patients with pathologically confirmed stage II or III gastric adenocarcinoma who underwent curative resection (plus D2 lymphadenectomy) in two tertiary hospitals from China between November 2010 and December 2020 were retrospectively reviewed. The demographic and clinicopathological data, operative variables, exact cycles of PAC and follow-up data were collected and carefully analyzed. The well-known CLASSIC study and some of our previous studies confirmed that patients receiving ≥ 6 cycles of chemotherapy had a significantly better prognosis [14–17]. Thus, patients undergoing 1 to 5 cycles of chemotherapy were considered to have incomplete PAC. The number of cycles of PAC was calculated as the sum of the number of cycles of NAC and AC. Patients who received no PAC, complete PAC (≥ 6 cycles), recurrent GC, with other synchronous malignancies, with missing essential medical data, lost to follow-up or died within 3 months after surgery were excluded from the study. This study was approved by the ethics committee of our institution (No. 114 in 2022). Written informed consent for operation and using their clinicopathological data was obtained from all patients prior to surgery.

### Peri-operative management and follow-up

Surgeons with sufficient experience performed or supervised all of the operations in accordance with the guidelines [4] and staged basing on the eighth edition of the Tumor-Node-Metastasis (TNM) system [18]. The overwhelming majority of stage II/III GC patients in our institutions received resection and AC, using XELOX or SOX regimens [14, 19]. Some patients with stage II diseases or those with stage III diseases but poor condition, single regimen (S-1 alone) were sometimes performed. About 15% with cT3-4/N + diseases underwent 1–5 cycles of NAC involving fluorouracil- and platinum-based regimens, such as ECF, FLOT, XELOX or SOX combinations [3, 7]. AC was usually started about 1 month after surgery and lasted for circa 6 months [14–15, 20].

Post-operative complications were diagnosed and classified according to the Clavien-Dindo system [21]. Patients were followed-up every 3 months in the initial 2 years, and then every 6 months from the 3rd to 5th year, and once a year thereafter, until December 2021. OS was calculated as months from the time of surgery to death or the last follow-up day, whichever occurred first.

### Statistical analysis

The entire cohort of patients were randomized in a 7:3 ratio into a training group or a validation group with a random seed. A χ^2^ test or Fisher’s exact test was used to compare categorical variables between groups and Student’s *t*-test was employed to test for differences between continuous variables. Kaplan-Meier curves and a log rank test were used to identify differences in OS. Data analyses were performed using R software (ver. 3.5.1, R Foundation for Statistical Computing) or SPSS ver. 24.0 software (IBM Corporation, NY, USA). All tests were bilateral and a *P*-value < 0.05 was considered to be a significant finding.

### Nomogram development and validation

The nomogram was built using the variables confirmed by multivariate Cox regression analyses in the training cohort. For each patient, the first line shows the exact point endowed for each factor loading on the predictor axis. After calculating the sum of the points, we could show the survival probability. Receiver operating characteristic (ROC) curves for the nomogram was generated basing on the area under the curve (AUC). The concordance index (C-index) was calculated to assess the performance of nomogram on training and validation groups, respectively. Calibration curves (1,000 bootstrap resamples) were plotted to assess the predictive power of the nomogram in the validation group. In addition, the decision curve analysis (DCA) was utilized to compare the clinical practicability between the nomogram and eighth version of TNM staging system.

## Results

### Baseline characteristics

As shown in Table 1, 1,070 consecutive patients were included in the present study. More than half the patients were male (66.1%), with stage III disease (72.4%), who underwent distal subtotal gastrectomy (67.1%) by an open procedure (75.2%). The mean body mass index (BMI) was 21.87 kg/m^2^ (range 13.84–36.63), mean age was 55.9 years (range 24–84), and the mean post-operative hospital stay was 11.8 days (range 4–79). A total of 101 patients (9.4%) developed ≥ grade II of post-operative complications. Patients received 1 to 5 cycles of PAC, with a median of 3 cycles. After randomization at a ratio of 7:3, 749 and 321 patients were separately enrolled into the training cohort or the validation cohort. As shown in Table 1, variables were all comparable between the 2 groups, except for the BMI. Patients in the training group had significantly higher BMI values than the validation group (21.98 vs. 21.60 kg/m^2^, *P* = 0.046).

**Table 1:** Clinicopathological characteristics of the training and validation cohort for patients with stage II/III gastric cancer (*n* = 1070)

Variables	Training cohort (*n* = 749 )	Validation cohort (*n* = 321)`	*P* value
Gender (males)	501 (66.9%)	206 (64.2%)	0.390
Age (years)	55.73 ± 10.05	56.03 ± 10.73	0.661
Body Mass Index (kg/m^2^)	21.98 ± 2.90	21.60 ± 2.82	0.046
Any comorbidities	217 (29.0%)	99 (30.8%)	0.539
Neo-adjuvant chemotherapy	68 (9.1%)	34 (10.6%)	0.440
Pre-operative albumin concentration (g/L)	38.73 ± 4.57	38.68 ± 4.98	0.868
Pre-operative lymphocyte count (×10 ^9^/L)	1.77 ± 0.71	1.79 ± 0.74	0.785
Pre-operative hemoglobin (g/L)	117.90 ± 25.00	118.07 ± 25.88	0.922
Operation method			0.699
Open	561 (74.9%)	244 (76.0%)	
Laparoscopy	188 (25.1%)	77 (24.0%)	
Type of resection			0.618
Distal subtotal gastrectomy	506 (67.6%)	212 (66.0%)	
Proximal subtotal gastrectomy	18 (2.4%)	11 (3.4%)	
Total gastrectomy	225 (30.0%)	98 (30.5%)	
T stage*			0.899
T1	22 (2.9%)	6 (1.9%)	
T2	79 (10.5%)	31 (9.7%)	
T3	100 (13.4%)	39 (12.1%)	
T4	548 (73.2%)	242 (75.4%)	
N stage*			0.415
N0	167 (22.3%)	61 (19.0%)	
N1	141 (18.8%)	68 (21.2%)	
N2	196 (26.2%)	77 (24.0%)	
N3	245 (32.7%)	115 (35.8%)	
pTNM stage*			0.086
II	218 (29.1%)	77 (24.0%)	
III	531 (70.9%)	244 (76.0%)	
Intra-operative blood loss (mL)	215.55 ± 145.25	208.03 ± 130.70	0.425
Operation time (min)	200.44 ± 52.78	204.29 ± 52.49	0.278
Peri-operative blood transfusion			0.655
Yes	156 (20.8%)	63 (19.6%)	
No	593 (79.2%)	258 (80.4%)	
Post-operative complications †			0.600
Yes	73 (9.7%)	28 (8.7%)	
No	676 (90.3%)	293 (91.3%)	
Post-operative hospital stays (days)	11.67 ± 5.00	12.09 ± 5.63	0.171
Peri-operative chemotherapy			0.880
1 cycles	133 (17.8%)	62 (19.3%)	
2 cycles	141 (18.8%)	66 (20.6%)	
3 cycles	148 (19.8%)	63 (19.6%)	
4 cycles	213 (28.4%)	84 (26.2%)	
5 cycles	114 (15.2%)	64 (19.9%)	

Data are presented as mean ± SD or n (%).

* Tumor stages are based on 8th edition of the Union for International Cancer Control TNM classification.

† Defined as Clavien-Dindo grade II or greater.

### Univariate and multivariate analyses

**Fig. 1:**
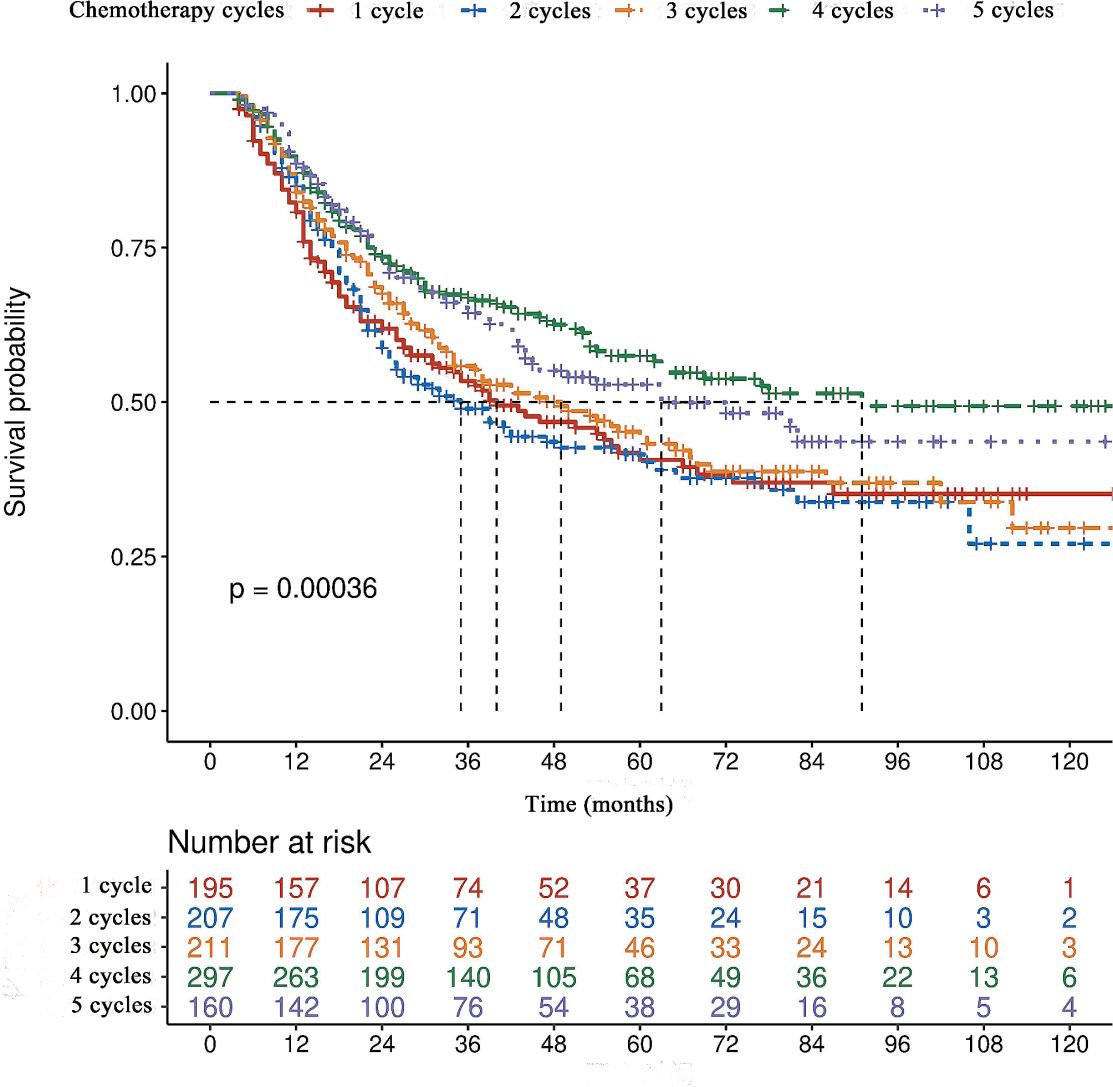
Overall survival curves of the entire 1070 patients who underwent curative resection for stage II/III gastric cancer stratified by the received cycles of peri-operative adjuvant chemotherapy (Compared by log rank test)

The median duration of follow-up was 30 months (range, 4–132) in the training group and 29 months (range, 4–128) in the validation group. As presented in Table 2, univariate analyses revealed that a lower BMI (< 18.5 kg/m^2^), higher intra-operative blood loss (≥ 300 mL), total gastrectomy, pTNM stage III disease, peri-operative blood transfusion and fewer cycles of PAC were all potential adverse factors related to poorer prognosis in the training cohort (all *P* < 0.05). After taking all of the factors with a *P*-value < 0.05 from univariate analyses in multivariate Cox regression analyses, only a lower BMI, total gastrectomy, stage III disease and fewer cycles of PAC were confirmed to be independent predictors for poorer survival. With respect to the number of cycles of PAC, the median OS in patients who received only 1 cycle was 37 months, which was comparable with that of 35 months and 45 months for patients who underwent 2 or 3 cycles, but significantly shorter than that of 91 months and 72 months for those who received 4 or 5 cycles of PAC, respectively (Fig. 1).

**Table 2:** Univariate and multivariate analyses of prognostic factors for overall survival following radical gastrectomy of stage II/III gastric cancer in the training cohort (*n* = 749)

Variables	N	Median OS time (months)	UV *P* value	MV HR (95% CI)	MV *P* value
Gender			0.287		
Male	501	54.0			
Female	248	47.0			
Age (years)			0.177		
≥ 65	147	45.0			
< 65	602	56.0			
Body mass index (kg/m^2^)			< 0.001		0.003
≥ 25.0	108	77.0		Reference	
18.5–24.9	566	53.0		1.296 (0.926–1.814)	
< 18.5	75	29.0		2.086 (1.356–3.208)	
ASA score			0.993		
≥ 3	58	56.0			
< 3	691	53.0			
Comorbidities			0.326		
Yes	217	67.0			
No	532	49.0			
Hemoglobin (g/L)			0.524		
≥ 100	581	54.0			
< 100	168	50.0			
Albumin level (g/L)			0.745		
≥ 35	608	56.0			
< 35	139	47.0			
Lymphocyte count (×10 ^9^/L)			0.744		
≥ 1.5	507	56.0			
< 1.5	242	52.0			
Operation procedure			0.152		
Open	561	50.0			
Laparoscopy	188	68.0			
Operation time (min)			0.080		
≥ 240	157	35.0			
< 240	568	56.0			
Intra-operative blood loss (mL)			0.003		0.128
≥ 300	170	34.0			
< 300	579	63.0			
Type of resection			< 0.001		< 0.001
Sub-total gastrectomy	524	82.0		Reference	
Total gastrectomy	225	23.0		1.921 (1.545–2.389)	
pTNM stage ^†^			< 0.001		< 0.001
II	218	NA*		Reference	
III	531	32.0		2.970 (2.215–3.982)	
Peri-operative blood transfusion			0.036		0.945
Yes	156	37.0			
No	593	56.0			
Post-operative complication ^‡^			0.314		
Yes	73	39.0			
No	676	56.0			
Peri-operative adjuvant chemotherapy			0.001		0.001
1 cycles	133	37.0		Reference	
2 cycles	141	35.0		1.103 (0.733–1.401)	
3 cycles	148	45.0		0.995 (0.722–1.373)	
4 cycles	213	91.0		0.549 (0.398–0.758)	
5 cycles	114	72.0		0.609 (0.422–0.880)	

ASA, American Society of Anesthesiologists; OS, overall survival; CI, confidence interval; HR, hazard ratio; UV, univariate analysis; MV, multivariate analysis; NA, not available.

* The median overall survival time has not reached during the follow-up.

† Tumor stages are based on 8th edition of AJCC TNM classification.

‡ Defined as Clavien-Dindo grade II or greater.

### Subgroup analyses

Among the 102 patients who were performed NAC, The median OS for patients receiving 1 cycle (*n* = 3), 2 cycles (*n* = 12), 3 cycles (*n* = 16), 4 cycles (*n* = 40) and 5 cycles (*n* = 31) of peri-operative adjuvant chemotherapy were 12, 17, 29, not available and 19 months, respectively. It seemed that patients underwent 4 cycles of chemotherapy had the best prognosis, and patients with 1 cycle of chemotherapy had the worst prognosis. But the difference was not statistically significant (*P* = 0.162, **Supplementary Fig.** 1).

### Survival predictive probability and accuracy

**Fig. 2:**
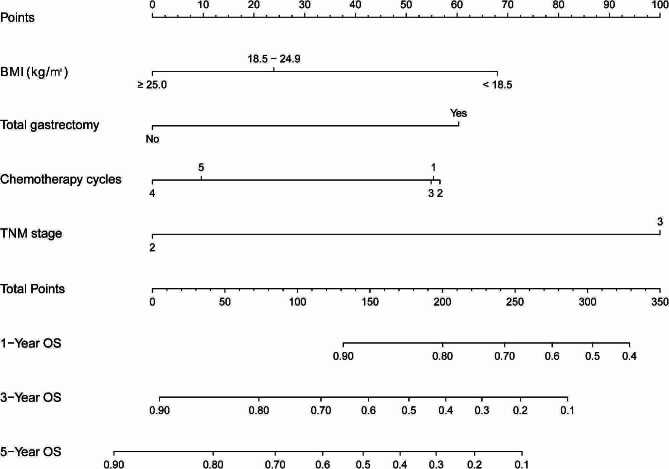
A nomogram for 1-, 3- and 5-year overall survival for stage II/III gastric cancer who underwent curative resection and incomplete peri-operative adjuvant chemotherapy (1–5 cycles)

According to the findings of multivariate Cox regression analyses performed in the training group, 4 independent predictors (BMI, total gastrectomy, TNM stage and PAC) were utilized to develop a nomogram to estimate the probability of survival (Fig. 2).

**Fig. 3:**
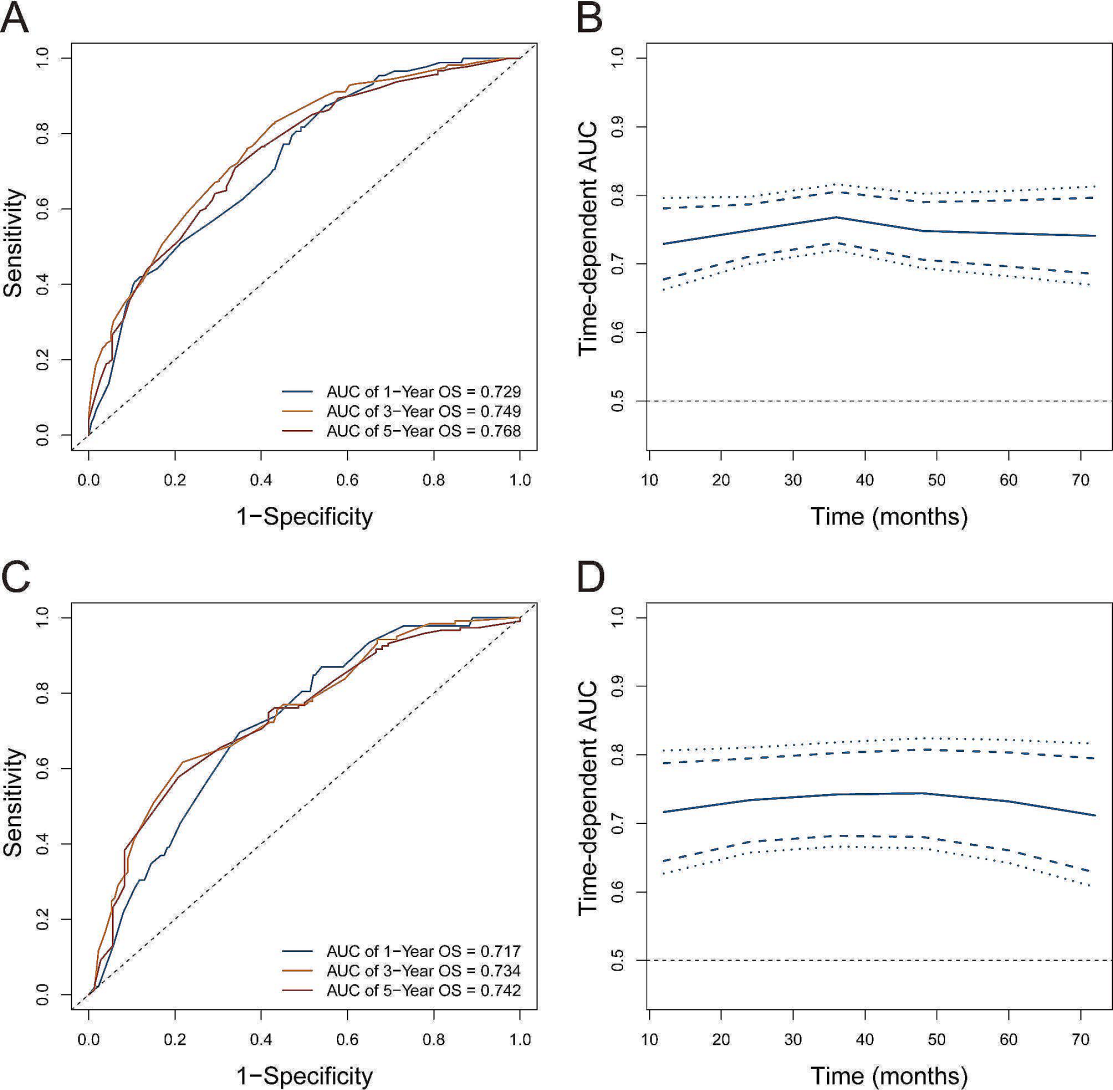
Receiver operating characteristic area under the curves of nomogram for predicting overall survival in the training (**A** and **B**) and validation (**C** and **D**) cohort

As shown in Fig. 3, in the training group, the AUC values to predict the 1- 3-, and 5-year survival probabilities were 0.729, 0.749, and 0.768, respectively, whereas the AUC values were 0.717, 0.734 and 0.742 in the validation group, separately. As a result, the developed nomogram using the 4 factors showed good predictive ability both for the training group and validation group. The C-index in the training and validation groups were 0.700 (95%CI: 0.674–0.726) and 0.689 (95%CI: 0.646–0.732), respectively.

**Fig. 4:**
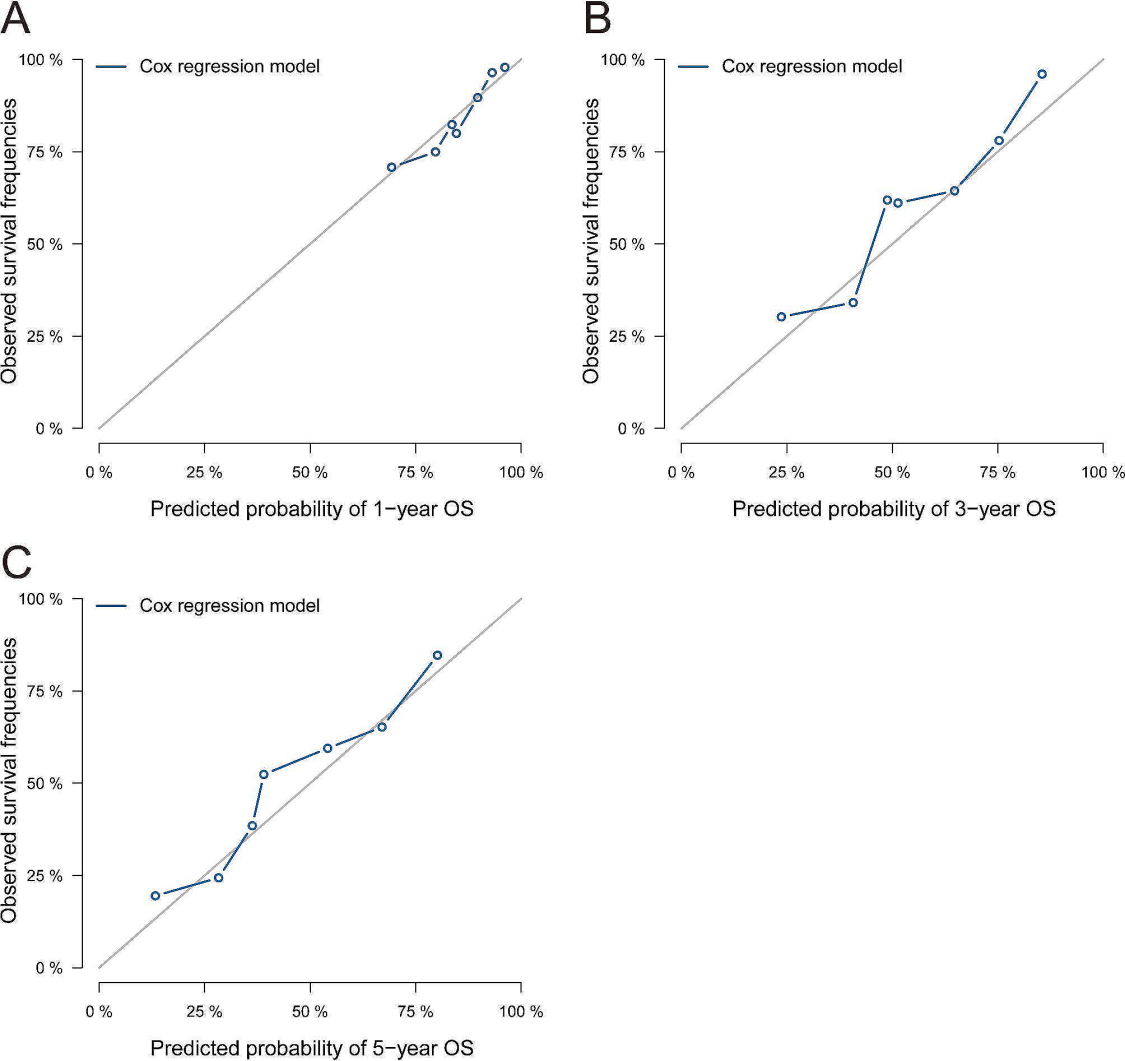
Calibration curves for the nomogram to predict (**A**)1-, (**B**)3- and (**C**)5- year overall survival for stage II/III gastric cancer who underwent curative resection and incomplete peri-operative adjuvant chemotherapy (1–5 cycles) in the validation cohort

**Fig. 5:**
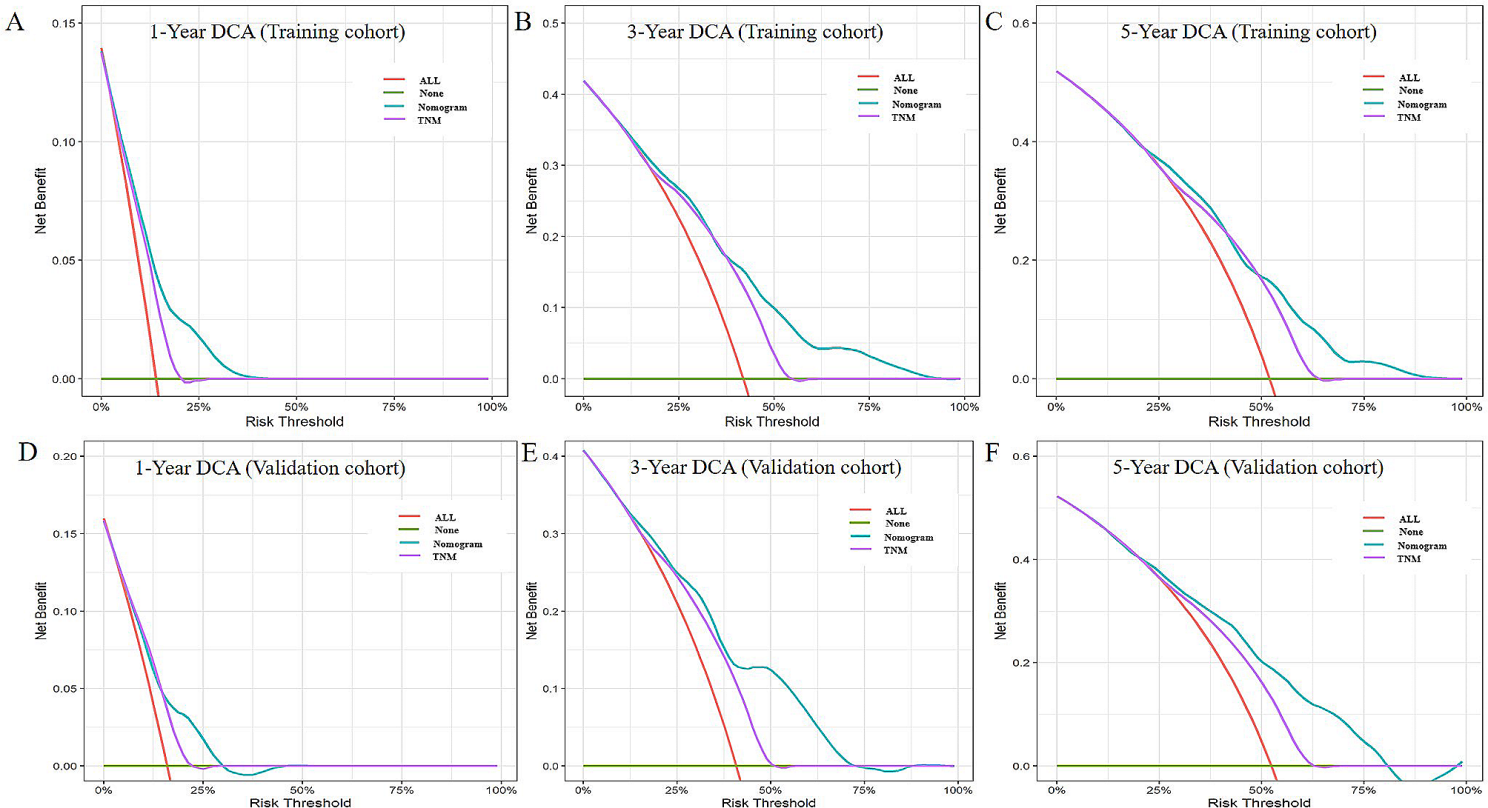
Decision curve analysis (DCA) of the nomogram. The DCAs of the nomogram in the training group (**A**-**C**) and the validation group (**D**-**E**) were plotted basing on the 1-, 3- and 5-year overall survival, respectively

Thereafter, the calibration curve was used to assess the discriminate the ability of the nomogram. Fig. 4 shows that the calibration curves revealed satisfactory consistence between the predicted and observed 1-, 3- and 5-year survival probabilities in the validation group. Additionally, DCA of the nomogram show a greater range of death risks within the net benefit than the eighth version of TNM staging system both in the training and validation groups (Fig. 5).

## Discussion

Although several nomograms have been established to forecast recurrence or the survival probability of GC patients [8, 10–11], none have focused on patients who received incomplete PAC (defined as 1–5 cycles in the present study). Moreover, whether the exact cycles of PAC was independently related to long-term outcomes has not previously been clarified. In this retrospective study of 1,070 consecutive patients with stage II/III GC who underwent radical gastrectomy and incomplete PAC in two tertiary hospitals in China, we confirmed that the number of chemotherapy cycles, along with BMI, total gastrectomy and stage III disease were significantly linked with prognosis. Thereafter, for the first time, a nomogram was constructed to predict survival probability, exploring the influence of the number of chemotherapy cycles in these patients. Further analyses established that the nomogram had satisfactory calibration and good discrimination to predict the 1-, 3- and 5-year survival probabilities. It seemed to be more consistent with clinical practice than the eighth version of TNM staging system.

PAC has been established as the most effective adjuvant management for LAGC patients [3–4], but it is common to encounter patients who could not complete all of the planned dose intensity. In one of our previous studies [16], we reported that among the entire 2,510 patients with LAGC, 546 patients (21.8%) underwent no AC and another 1,044 patients (41.6%) received 1 to 5 cycles of PAC. Only 920 patients (36.7%) underwent ≥ 6 cycles of PAC. Not surprisingly, patients with adequate PAC (≥ 6 cycles) had significantly better prognosis. It was echoed by Lu and colleagues [9], who reported that 425 patients (25.6%) among a cohort of 1,657 did not receive even one cycle of AC in a retrospective multicenter study. Further analyses confirmed that AC significantly decreased recurrence but the exact cycles of AC were not reported. More importantly, in the well-known CLASSIC study [14], 174 of 520 patients (33.5%) with stage II-IIIB GC could not complete the planned 8 cycles of adjuvant oxaliplatin and capecitabine therapy. Post hoc analysis identified that patients who received at least 6 cycles had significantly prolonged survival than those with low dose intensity. Another of our previous studies also confirmed that patients who were given ≥ 6 cycles of PAC had significantly better cancer-specific survival (CSS) [15]. Thus, patients undergoing 1–5 cycles were defined to have incomplete PAC in the present study. As shown in Table 2, the prognosis was comparable in patients undergoing 1, 2 or 3 cycles of PAC, but significantly worse than those who underwent 4 or 5 cycles of PAC. Consistence with previous studies, our findings confirmed the relationship between dose intensity and prognosis, and thus, emphasized the importance of completing as many PAC cycles as possible in stage II/III GC patients [9, 14–16].

The inclusion criteria, investigated variables and conclusions differed significantly in predictive models for GC patients. Liu et al. [8] established a nomogram to predict CSS in 688 stage II or III patients who had received more than 4 cycles of AC after resection. They concluded that inflammatory, nutritional and tumor markers, tumor location and the stage were significant predictors for CSS. The C-index of their nomogram based on these factors was 0.714, which was higher than the TNM stage (0.630, *P* < 0.001). Park and colleagues [10] developed a new staging system and a nomogram for advanced GC patients without adjuvant managements. They confirmed that post-operative morbidity, age and American Society of Anesthesiologists (ASA) score were independent predictors for OS. However, it should be noted that only 185 patients were included in the training group. In another retrospective study that included 639 stage I to III GC patients (except T1a) who underwent ≥ 2 cycles of AC within 2 months following their operations, single chemotherapy regimens were identified to be associated with a poorer prognosis compared to multiple chemotherapy regimens [11]. Unfortunately, the exact number of cycles of AC was not given. In addition, there has been increasing evidence favoring PAC instead of AC for the treatment of locally advanced GC, even in Eastern countries [7, 19]. It is noteworthy that almost all previous similar studies excluded those involving the administration of NAC, which inevitably harmed the generalizability of the conclusions [8–9, 11].

The prognostic factors and recurrence patterns might be different among patients given different dose intensities. In the study presented by Kanda et al. [21], 70 pairs of stage II/III patients were analyzed after propensity score matching. The authors identified that stage III, pT4, vessel invasion, total gastrectomy and carcinoembryonic antigen levels ≥ 5 ng/mL were independent predictors for long term survival in patients given no AC, whereas only a macroscopic tumor size ≥ 5 cm was significantly associated with survival in patients who received adjuvant S-1. In our previous studies, we have confirmed that complete PAC (≥ 6 cycles) could cancel out the adverse influence of a low prognostic nutritional index, low BMI, peri-operative blood transfusion and/or infections on survival in stage II or III GC patients [15–17]. Given that PAC could impact recurrence patterns and modify the predictive factors for survival in GC patients [22–23], we must cite the previous conclusions with caution and a new nomogram was clearly needed in patients treated incompletely with PAC.

Consistent with our previous findings, we also revealed that a poor nutritional status, late TNM stage and total gastrectomy were significantly related to poor prognosis [8–9, 11, 16–17, 22]. In addition, the exact dose intensity was confirmed to be an independent predictor of prognosis for the first time. Previous studies revealed that poor patient condition (age ≥ 65 years, ASA score ≥ 3), a poor nutritional and immune status (serum albumin level < 35 g/L, prognostic nutritional index < 43.9, BMI < 20.3 kg/m^2^), body weight loss and post-operative infection complications adversely affected compliance with AC [15, 24–27]. The possible explanations included that poor patient condition and nutritional status increased chemotherapy-related adverse events [28]. Post-discharge oral nutritional supplements significantly decreased weight loss and chemotherapy modifications after 3 months of invention [29]. On the other hand, NAC was confirmed to be a protective factor for patients to complete PAC, especially in those at a high risk of experiencing post-operative complications [16, 30]. Taken together, the findings suggest that in order to increase the completeness of PAC and improve prognosis, performing NAC and nutritional intervention are feasible strategies. But further prospective studies are still needed.

The present study had several limitations. First, it was a retrospective study and some important variables, such as tumour markers, the type of chemotherapy regimen, the exact time to initiate treatment, the exact number of patients having dose de-escalation, changing chemotherapy regimen or were put on single agent treatment was not collected and analyzed, which might also act as confounders and impact the reliability of our conclusions. Second, given that it was a long study period over 10 years, several chemotherapy regimens combinations were used in our institutions, such as S-1 alone, ECF, FLOT, XELOX and SOX [17]. The convenience and safety of different regimens might also impact the completion of PAC. Third, the median follow-up of 30 months of the entire cohort seemed not long enough to analyze later relapse and the deaths of patients. Fourth, for the 102 patients (9.5%) undergoing NAC, the pre-treatment clinical TNM stage was used, which might be inconsistent with the pathological TNM stage. Fifth, given that only a small proportion of patients underwent laparoscopic surgery, whether the conclusions were applicable to these patients still needs further investigation. Last but not least, our findings still need external validation, especially in patients from Western countries, where NAC is recommended as standard treatment for LAGC and the management strategy differed significantly from our institutions [3, 30].

## Conclusions

A nomogram has been constructed to predict the survival probability of patients with stage II or III GC who underwent curative resection and incomplete PAC, using a large sample size of patients from two tertiary hospitals in China. We also explored the influence of exact cycles of PAC and confirmed that the dose intensity could have a significant impact on long term outcomes. Further analyses showed that the nomogram incorporating BMI, total gastrectomy, TNM stage and PAC variables produced a satisfactory calibration and good discrimination. Our findings should assist clinicians to evaluate the survival probability of patients receiving incomplete PAC, and encourage patients to complete all of the planned chemotherapy regimens.

### Electronic supplementary material

Below is the link to the electronic supplementary material.

Supplementary Material 1

## Data Availability

The datasets used and/or analyzed during the current study are available from the corresponding author on reasonable request.
