# Supplementary material for: Nomogram to predict overall survival of patients receiving radical gastrectomy and incomplete peri-operative adjuvant chemotherapy for stage II/III gastric cancer: a retrospective bi-center cohort study
Source: BMC Cancer. 2024 Mar 18;24:344. doi: 10.1186/s12885-024-12103-1 (PMC10946121; doi:10.1186/s12885-024-12103-1)

**Supplementary Figure 1.** Overall survival curves of the 102 patients who underwent neo-adjuvant chemotherapy and curative resection for stage II/III gastric cancer stratified by cycles of chemotherapy (Compared by log rank test).


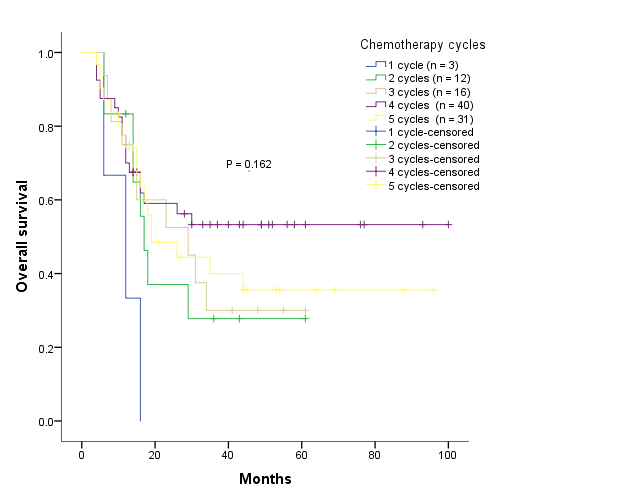

Supplement: Supplementary file 1 — Supplementary Material 1 [file 12885_2024_12103_MOESM1_ESM.doc]
